# Understanding perceptual decisions by studying development and neurodiversity

**DOI:** 10.1177/09637214231162369

**Published:** 2023-04-16

**Authors:** Catherine Manning, Gaia Scerif

**Affiliations:** a School of Psychology and Clinical Language Sciences, University of Reading, UK; b Department of Experimental Psychology, University of Oxford, UK

## Abstract

A cornerstone of human information processing is how we make decisions about incoming sensory percepts. Much of psychological science has focused on understanding how these judgements operate in skilled adult observers. While not typically the focus of this research, there is considerable variability in how adults make these judgements. Here, we review complementary computational modelling, electrophysiological data, eye-tracking and longitudinal approaches to the study of perceptual decisions across neurotypical development and in neurodivergent individuals. These data highlight multiple parameters and temporal dynamics feeding into how we become skilled adult perceptual decision makers, and which may help explain why we vary so much in how we make perceptual decisions.

As skilled adult observers, we readily make accurate and fast decisions about sensory inputs of many different kinds, from dynamically changing moving stimuli to highly complex and cluttered visual scenes. Parameters underpinning optimal adult perceptual decision making have been the focus of much research in psychological science. A perhaps less well understood question is how we come to differ from each other in making such decisions. Here, we outline evidence from complementary approaches in the developmental cognitive neurosciences that point to the need to understand differences at multiple time-points of divergence in the complex dynamics leading to optimal perceptual decision making. Computational modelling, temporally sensitive neuroimaging methods and eye-tracking provide rich data on how diversity in perceptual decision making emerges, for both neurotypical and neurodivergent individuals. We begin with evidence that, when comparing children and adults, parameters feeding into perceptual decisions differ across age groups, from the onset of stimulus processing, taking moving stimuli as a central case in example. We then move onto developmental differentiation in the period during which we prepare to make perceptual decisions on the basis of our goals, prior knowledge or information recently committed to memory. We conclude by overviewing evidence from autism, dyslexia and genetic syndromes, suggesting multiple influences and routes to perceptual decision-making dynamics, both before and after the onset of incoming stimuli.

## 1 The developmental dynamics of perceptual decisions: The case of motion processing

Perceptual decisions have commonly been studied in adults using random-dot motion tasks. These tasks allow the strength of sensory evidence to be manipulated precisely, and the neurophysiological correlates of sensory encoding and decisional processes have been disentangled in non-human animals or in human adults performing these tasks (Kelly & O’Connell, 2015), making them useful for deconstructing multiple processes contributing to decisions. However, children provide a very interesting and far less explored model in which to study the emergence of decision parameters, because they show a protracted development of sensitivity to motion information, with adult-like levels of sensitivity not being reached until mid-to-late childhood (Hadad et al., 2011; Manning et al., 2014) and. So far, most developmental studies have focused on characterising the underlying sensory parameters affecting performance (Hadad et al., 2015; Meier & Giaschi, 2014), rather than considering developmental changes in the multiple cognitive processes underlying perceptual decisions. Yet, by applying complementary methods such as modelling and electroencephalography, as well as a developmental perspective, we can uncover multiple processes that contribute to efficient adult perceptual decisions.

For example, Manning et al. (2014) used a computational modelling technique to investigate contributors to age-related differences in motion processing ability. They studied whether the development of motion coherence sensitivity is limited by internal noise (i.e., imprecision in estimating the directions of individual elements) and/or global pooling across local estimates, by presenting moving dots under equivalent noise direction discrimination conditions and in motion coherence conditions, at both slow (1.5°/s) and fast (6°/s) speeds to children aged 5, 7, 9 and 11 years, and adults. As children got older, their levels of internal noise reduced, and they were able to average across more local motion estimates, but age-related improvements in coherent motion perception were predicted by improvements in averaging, and not by reductions in internal noise. In other words, improved performance in older children and adults was driven by how efficiently they combined motion signals over space, rather than how precisely they estimated the direction of individual moving elements in a visual scene.

**Figure 1:**
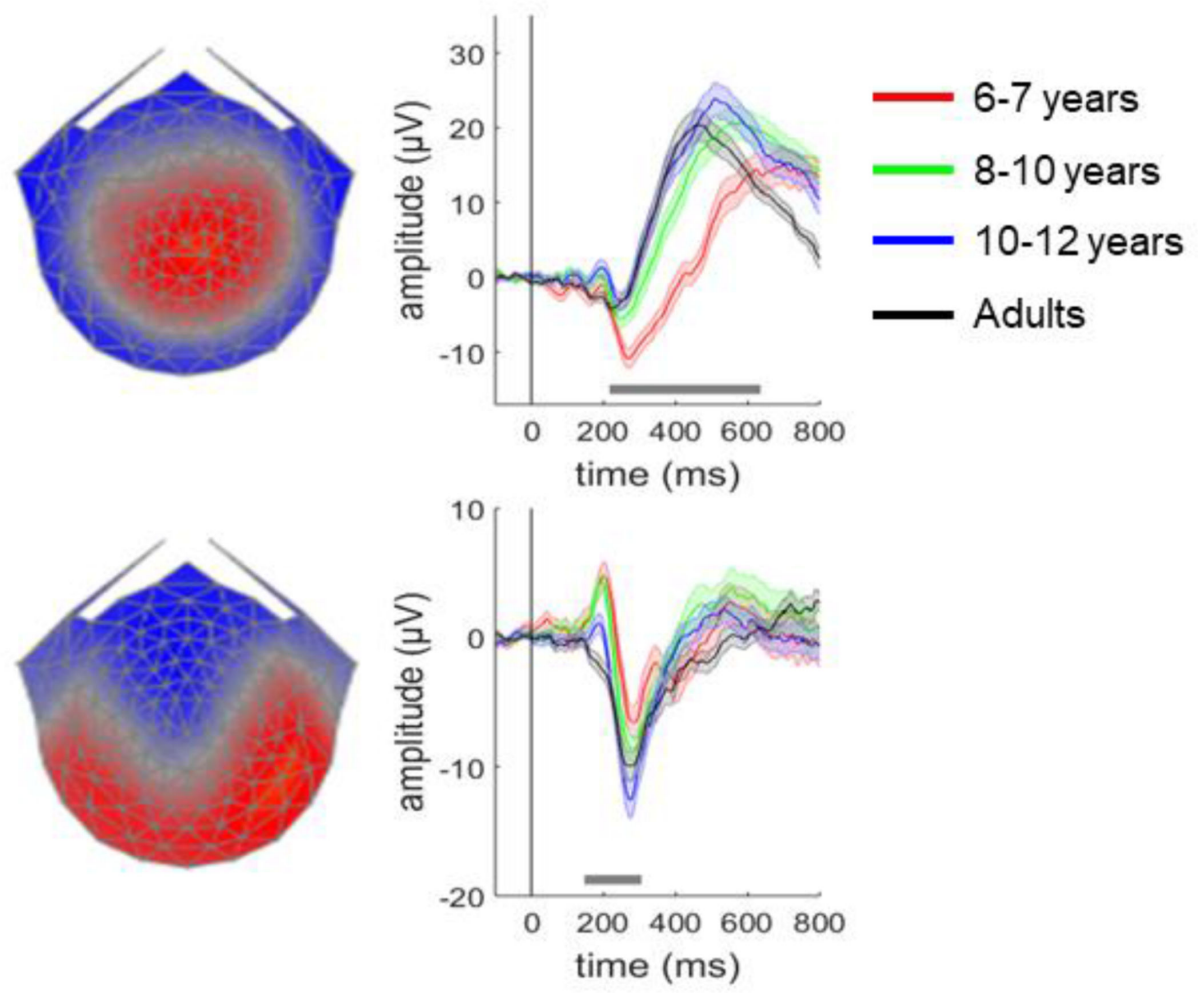
Age-related differences in EEG activity during perceptual decision-making tasks. Manning et al. (2019) extracted two patterns of EEG activity (‘components’) when adults were making perceptual decisions about the overall direction of moving dots: a sustained component with maximal activity over centro-parietal electrodes (over the middle of the head) which gradually increased over time (upper left panel) and an earlier component with maximal activity over occipital electrodes (at the back of the head; lower left panel). Following previous work, these components were linked to the decision-making process and early sensory encoding of motion information, respectively. As shown in the rightward panels, the activity within both components showed age-related differences in amplitude and shape, with horizontal grey bars highlighting areas of significant differences between age groups. These results suggest that age-related changes in perceptual decisions are accompanied by both changes in early processes linked to sensory encoding and later decision-making processes. Adapted from Manning et al. (2019).

**Figure 2:**
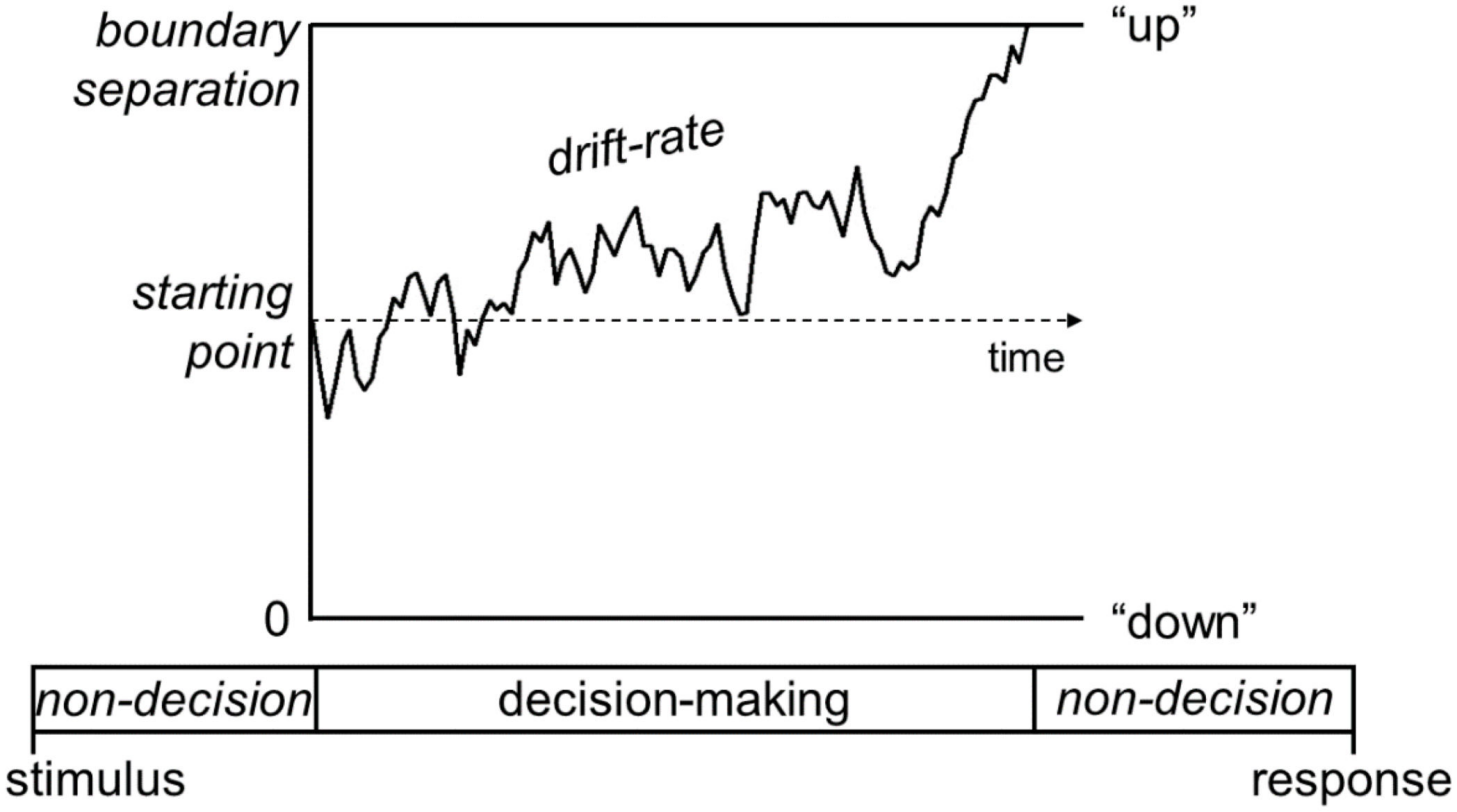
Schematic representation of the decision-making process in the diffusion model for a task that involves deciding between upwards and downwards motion. In the diffusion model, the decision-making process is represented as a noisy accumulation of evidence from a starting point towards one of two decision bounds. When a participant decides between upwards and downwards motion, the decision bounds correspond to “up” and “down” responses. In this representation, the overall motion in the stimulus is going upwards. Boundary separation represents the width between the two bounds and reflects how cautious an observer is. Wider decision boundaries reflect that more evidence is required before making a decision (i.e., more cautious responses). Drift-rate reflects the rate of evidence accumulation, which depends on both the individual’s sensitivity to a stimulus and the stimulus strength. Non-decision time is the time taken for sensory encoding processes prior to the decision-making process and response generation processes after a bound is reached. Manning et al. (2022a) showed that younger children accumulate evidence more gradually (i.e., have a lower drift-rate) and make decisions more cautiously (i.e., have wider decision bounds) than older children and adults, as well as taking longer for non-decision processes (i.e., longer non-decision time). Adapted from Manning et al. (2022b).

Additional insights into the dynamic evolution of processes contributing to perceptual decisions can be gained from electroencephalography (EEG), due to its high temporal resolution. We adapted for children and optimised for EEG a traditional motion coherence task. One hundred white stimulus dots (described to the children as fireflies) were randomly positioned within a central square region on a black screen and moved at a speed of 6°/s. The dots had a limited lifetime of 200 ms (with randomised starting lifetimes), and dots moving outside the square stimulus region were wrapped around to the opposite side. Each trial consisted of a fixation period, a random motion period, a stimulus period, and an offset period. The fixation period, during which only the central fixation square was shown, was presented for a randomly selected duration between 800 and 1000 ms. The stimulus dots first appeared in the random motion period, during which they moved in random, incoherent directions, for a randomly selected duration between 800 and 1000 ms. In the stimulus period, a proportion of the dots moved coherently either upward or downward (described as bees to be caught by a bee-keeper), while the remainder of the dots continued to move in random directions. The stimulus period lasted until a response was made, or until 2500 ms had passed. Finally, an offset period continued the coherent stimulus presentation for a randomly selected duration between 200–400 ms. The jittered durations of the fixation, random motion and offset periods were intended to minimise expectancy effects. While children and adults performed on this motion coherence task, we collected high-density EEG data from all participants and we showed that age-related differences in motion sensitivity are accompanied by differences in both a late, sustained decision-related component and an earlier component reflecting sensory encoding (as shown in Fig. 1, adapted from Manning et al. 2019). The late decision-related component is separable and distinguishable from upstream sensory signals: it predicted response time even when the strength of evidence was held constant, as well as scaling with motion coherence and increasing as a function of time (criteria set out by Kelly & O’Connell, 2013). The other component did not show the association with reaction time (Manning et al., 2019). In addition, as discussed in greater detail later, when we used an EEG processing approach geared to deconvolving stimulus-locked and response-locked signals (Manning et al., 2022 a and b), the late decision-related component in the deconvolved EEG signal correlated with drift rate, suggesting that it quite specifically indexes evidence accumulation, rather than for example, boundary separation or sensory / motor processes. These results suggest that there are multiple and separable signatures of the dynamics at which perceptual decisions take shape and change from childhood to adulthood. Complementing these results, we highlighted how multiple parameters differentiate younger children from older children and adults using diffusion modelling (Manning, Wagenmakers et al., 2021) – a cognitive model which breaks accuracy and response time into distinct processing stages (Ratcliff & McKoon, 2008; Fig. 2). Specifically, younger children had lower drift rates (reduced sensitivity), wider boundary separation (increased response caution) and longer non-decision times than older children and adults. Moreover, the slope of an EEG component that was maximal over centroparietal electrodes was steeper in adults compared to younger children (Manning et al., 2019) and it correlated positively with drift rate (Manning et al., 2021) suggesting that evidence accumulation changes over age are accompanied by changes in a response-locked EEG component.

By taking a developmental perspective that leverages similarities and differences between children and adults, the complementary approaches discussed above have significantly advanced our ability to unveil the multiple processes building to perceptual decisions from stimulus onset to response generation. These approaches can extend even earlier in the timeline of processing, to investigate the dynamics that precede incoming sensory stimuli and prepare an observer to make perceptual decisions.

## 2 Preparing to make perceptual decisions: The influence of prior history

In addition to the temporal dynamics and multiple parameters building to the decision point from stimulus onset, the decisions we make are also influenced by how we prepare to make those decisions, because of attentional biases from information we have encountered and committed to memory. This is for example the case when we have learnt over time that stimuli for which we are preparing to make perceptual decisions occur at particular locations in complex visual scenes, whether static or dynamic. Attentional biases that depend on information recently encoded into memory (“memory-guided attention” henceforth) influence incoming percepts, so that our prior history guides perceptual decision making (Nobre & Stokes, 2019). Again, a multi-method developmental cognitive neuroscience approach highlights multiple factors contributing to how we prepare to make perceptual decisions.

Doherty et al. (2017) combined computational approaches, eye-tracking methodology, and individual-differences measures, by asking participants to search for perceptual targets in complex visual scenes containing social or non-social distractors, and repeating exposure to those scenes and targets over blocks, to induce a learning history for their association. Eye-tracking revealed significantly more attentional capture to the social compared to the non-social distractors embedded in each scene. Critically, participants’ memory precision for target locations was poorer for social scenes compared to non-social ones. The difference between social and non-social scenes was moderated by individual differences in social anxiety, with anxious individuals remembering target locations better under conditions of social distraction than non-anxious individuals, suggesting an interplay between attentional dynamics during learning about the scenes, memory for those scenes and the characteristics of diverse observers, a point to which we return in section 3. Complementing this paradigm with EEG data collection, Doherty, van Ede et al. (2019) showed that, while they were preparing to make decisions about targets that had appeared in the context of social scenes, participants’ brain signatures of preparation differed for social vs. non-social targets. A lateralised modulation of alpha-band oscillations over one hemisphere compared to the other, a signature of preparatory EEG that is characteristic of attention while preparing to make perceptual decisions, was different when preparing to perceive targets that had been encoded into memory in the context of social scenes, compared to non-social scenes. These findings highlighted that preparing to make perceptual decisions differed depending on the context in which targets had appeared, suggesting a dynamic interplay between memory-guided attention, the nature of visual scenes and perceptual decisions about targets embedded in those scenes.

Investigating memory-guided attention via a developmental science perspective has further nuanced our understanding of adult perceptual decisions. Doherty, Fraser et al. (2019) asked six-toten year-old children and young adults to search for targets in complex visual scenes (following Doherty et al., 2017), while eye-tracking and simple manual responses were collected. Social stimuli distracted both children and adults during visual search, and eye-tracking revealed even greater attentional capture by social distractors for children compared to adults. Intriguingly, however, children demonstrated overall better memory precision for target locations than adults. Children spent more time inspecting the whole scene before reporting target locations during the learning phase. We hypothesize that this led to greater exploration of and encoding of the full scene during the longer time inspecting it. This slower performance would normally class as less efficient search performance, but this resulted in more precise later memory, suggesting retrieval of more precise target locations either via accessing scene gist and / or landmarks within it. A further replication, combining memory precision data with eye-tracking data collected not only during the learning phase, but also the memory phase, would test this proposed mechanism more directly. Finally, when participants detected targets within visual scenes, adults were slower for targets appearing at unexpected locations within social scenes compared to non-social scenes, but this was not the case for children, again perhaps pointing to better learning of the scene as a whole. In contrast, fast processing adults focused on locating targets are very likely to have suffered from more shallow processing of the highly salient and attention-grabbing social distractors. In other words, temporal search patterns that are “slow and exploratory” for children, rather than “fast and goal-driven” for adults may reduce interference from salient distractors during the attention orienting phase. Using a similar memory-guided attention paradigm, Nussenbaum, Scerif and Nobre (2019) also found evidence that children are better able than adults to draw from information encoded into memory when preparing to detect perceptual targets in complex visual scenes. Following on this work on memory-guided attention development, Shalev, Boettcher, Scerif and Nobre (2022) recently found that children as young as four years of age are as sensitive as adults to guiding attention to spatiotemporal regularities in dynamically changing scenes.

As a whole, these complementary data suggest that the interplay between attentional biases, memory and memory-guided attention is complex, changing over developmental time and moderated by individual differences across observers. In the next section, we turn precisely to how studying neurodiversity can inform our understanding of perceptual decisions and their dynamics.

## 3 Neurodiversity

When studying neurodiversity, the complex cascade of processes feeding into perceptual decisions is even more apparent. This is because differences at multiple stages of processing can converge or distinguish individuals with diverse profiles of development. Autism, which affects social communicative and non-social behaviours, and dyslexia, which affects reading ability, are distinct conditions that have both been linked to atypical motion sensitivity (Benassi et al., 2010; van der Hallen et al., 2019). Multi-method approaches can shed light on where differences lie, in autistic and dyslexic observers compared to neurotypical observers, and to identify areas of overlap and divergence between the conditions. Toffoli et al. (2021) investigated the temporal dynamics of EEG signals locked to the onset of directional motion stimuli and found that autistic and dyslexic children did not differ significantly from typically developing children in early stages of processing associated with early sensory encoding (in a component like that shown in Figure 1, lower panel). Yet, both autistic and dyslexic children differed from typically developing children in later stages of processing, thought to involve processes such as segregation of signal from noise and decision-making.

Despite these similar patterns in EEG activity for autistic and dyslexic children, diffusion modelling studies combined with response-locked EEG indices have uncovered differences in processing between autistic and dyslexic children. Dyslexic children show reduced accumulation of motion evidence compared to typically developing children, across two motion tasks that differed in their requirements for segregating signal from noise (Manning et al., 2022b). These differences were related to reductions in a response-locked EEG measure reflecting the decision-making process. Yet, a comparable study (Manning, Hassall et al., 2022a) showed no clear differences between autistic children and typically developing children in diffusion model parameters or the response-locked EEG measure. Future work is therefore needed to establish the conditions under which autistic children make atypical perceptual decisions about motion stimuli, and why this is the case. There is also some evidence that ADHD symptoms – in particular hyperactivity symptoms – might relate to perceptual decision-making parameters in autistic participants (Manning, Hassall et al., 2022a). Therefore, by applying multi-method approaches, we can identify both areas of overlap and areas of difference in how neurodivergent individuals make perceptual decisions.

Of note, further valuable insights can come from neurodivergent individuals for whom symptoms of autism and attention differences cluster together. Fragile X syndrome (FXS) is the most common genetically identified and inherited monogenic syndrome associated with a high prevalence of autistic and ADHD symptoms (Doherty & Scerif, 2017). By tracking autism and ADHD symptoms longitudinally over the course of three years across a diverse group of children and teenagers with FXS, Doherty et al. (2020) found that individual variation in ADHD symptoms predicted later individual variation in autism symptoms, bolstering the need to investigate more precisely how attention, activity levels and autistic characteristics interact over developmental time. Indeed, Guy et al. (2020) complemented the longitudinal approach by Doherty et al. (2020), by using the experimental memory-guided attention paradigm described in section 2, and by measuring the temporal dynamics of processing over time using eye-tracking in both neurotypical children and young people, as well as children and young people with FXS. The study revealed differences in the dynamics of attention to social stimuli for children and young people with FXS compared to neurotypical participants: children and young people with FXS inspected the social elements of complex visual scenes to a lesser degree than neurotypical individuals when they were first presented with them. This avoidance pattern decreased with experience of the scenes, but it was coupled with poorer memory for these scenes, again pinpointing the interplay between memory-guided preparation, stimulus processing and perceptual decisions.

In their entirety, complementary multi-method data about how neurodivergent children and young people make perceptual decisions converge in suggesting that there are multiple ways in which observers can differ over the timeline leading to perceptual decisions: from preparation, to processing of stimuli and decision-making, as well as in the short-term prior history and longitudinal change. Of note, we began by describing how a decision-theoretic framework inform can help disentangle distinct contributors to perceptual choice, and how these parameters manifest in temporally distinguishable differences between neurotypical and neurodivergent children (e.g., slower drift rate for some neurodivergent children). A parallel set of findings points again to dynamics, suggesting that preparatory and memory-dependent dynamics influence perceptual decisions differently across ages. However, the two literatures have not yet been bridged: i.e., to our knowledge developmental scientists have not yet tested whether pre-stimulus dynamics (preparation, memory-dependent attention effects) change post-stimulus decisional parameters (e.g., drift-rate, decision bounds), rather than simply affect perceptual choices. What is also not known is whether preparatory dynamics influence decisional parameters differently in neurotypical and neurodivergent children. Future research could integrate approaches to pre-stimulus and pre-decision dynamics, for both neurodivergent and neurotypical development from childhood into adulthood.

## 4 Conclusion

We have reviewed evidence that multiple complex parameters contribute to perceptual decisions, and that these parameters can differ in informative ways across development and with neurodiversity. A better understanding of the developmental processes involved may help us to in turn understand the perceptual decisions made by skilled adult observers, and in particular, why there is considerable variability in how people make perceptual decisions.
